# Supplementary material for: A 100 bp GAGA motif-containing sequence in AGAMOUS second intron is able to suppress the activity of CaMV35S enhancer in vegetative tissues
Source: PLoS One. 2020 Mar 5;15(3):e0230203. doi: 10.1371/journal.pone.0230203 (PMC7058354; doi:10.1371/journal.pone.0230203)
Supplement: Attachment — Submitted filename: Response to Reviewers.docx [file pone.0230203.s004.docx]

**Point to point response**

**Reviewer #1:**

I appreciate the efforts by the authors to address my concern. I am satisfied. Could the author integrate some of the responses they gave in the rebuttal into the manuscript? For example, more details about the potential application of the findings.

**Response:** Thanks a lot for your kind comments and valuable suggestions. We have integrated some information in the reponse into the revised manuscript. In the Introduction of the revised manuscript (Page 2, Paragraph 1, Line 40-44), we explained the merit of the synthetic promoters. In the Discussion of the revised manuscript (Page 9, Paragraph 4, Line 349-351), we summarize the potential application of the findings in the plant biotechnology and in the investigation of *AGAMOUS* gene in plants.
